# A Prognostic Model Based on the Log Odds Ratio of Positive Lymph Nodes Predicts Prognosis of Patients with Rectal Cancer

**DOI:** 10.1007/s12029-024-01046-2

**Published:** 2024-05-03

**Authors:** Jian Li, Yu zhou Yang, Peng Xu, Cheng Zhang

**Affiliations:** 1 Department of General Surgery, General Hospital of Northern Theater Command (Teaching Hospital of China Medical University), Shenyang, China; 2 Jinzhou Medical University, Jinzhou, China

**Keywords:** Colon cancer, LODDS, SEER, Nomogram, Survival analysis

## Abstract

**Objective:**

This study aimed to compare the prognostic value of rectal cancer by comparing different lymph node staging systems, and a nomogram was constructed based on superior lymph node staging.

**Methods:**

Overall, 8700 patients with rectal cancer was obtained from the Surveillance, Epidemiology, and End Results (SEER) database between 2010 and 2015. The area under the curve (AUC), the *C* index, and the Akaike informativeness criteria (AIC) were used to examine the predict ability of various lymph node staging methods. Prognostic indicators were assessed using univariate and multivariate COX regression, and further correlation nomograms were created after the data were randomly split into training and validation cohorts. To evaluate the effectiveness of the model, the *C* index, calibration curves, decision curves (DCA), and receiver operating characteristic curve (ROC) were used. We ran Kaplan-Meier survival analyses to look for variations in risk classification.

**Results:**

While compared to the N-stage positive lymph node ratio (LNR), the log odds ratio of positive lymph nodes (LODDS) had the highest predictive effectiveness. Multifactorial COX regression analyses were used to create nomograms for overall survival (OS) and cancer-specific survival (CSS). The *C* indices of OS and CSS for this model were considerably higher than those for TNM staging in the training cohort. The created nomograms demonstrated good efficacy based on ROC, rectification, and decision curves. Kaplan-Meier survival analysis revealed notable variations in patient survival across various patient strata.

**Conclusions:**

Compared to AJCC staging, the LODDS-based nomograms have a more accurate predictive effectiveness in predicting OS and CSS in patients with rectal cancer.

**Supplementary Information:**

The online version contains supplementary material available at 10.1007/s12029-024-01046-2.

## Introduction

Rectal cancer makes up around 40% of colorectal cancer cases, which is a frequent malignant tumor of the digestive tract that ranks second in death and the third in incidence among all malignant tumors [1]. The prognosis of patients with rectal cancer and the necessity for adjuvant therapy are significantly influenced by the existence of lymph node metastases [2–4]. Currently, The American Joint Committee on Cancer (AJCC) staging system is primarily used to stage lymph nodes; however, the anatomy of the pathologist, the technique of the surgeon, tumor heterogeneity, and adjuvant treatments (chemotherapy and radiation) all have an impact on the lymph nodes, which in turn affects the patient’s tumor staging and causes the phenomenon of staging migration [5, 6], and thus affects the treatment and prognosis of patients.

The lymph node ratio (LNR) and the log odds ratio of positive lymph nodes (LODDS) have been shown in multiple studies to have significant prognostic value in various tumors [7–11] and can partially offset the lack of lymph node count, which can alleviate the under or overstatement of the conventional N staging, which is based on the number of a single positive lymph node, and achieve accurate staging and improved clinical outcome for patients with rectal cancer [12]. There are stringent restrictions on the pathology and pathological staging of the patients included in the existing research based on LNR or LODDS, and these studies are not generally applicable. A specific assessment of the various risk stratifications for adjuvant therapy is also lacking, and the usefulness of the lymph node staging threshold still needs to be determined.

Nomograms are now commonly used to assess the prognosis of tumors. They do this by thoroughly evaluating each patient’s various prognostic variables and risk factors, providing individualized prognostic analysis for each patient, improving prediction accuracy, and yielding numerous advantages over traditional TNM staging [13, 14]. Numerous prognostic research on rectal cancer makes use of it.

The majority of the predictive nomograms now in use for rectal cancer have not been further examined for adjuvant therapy and have not been able to overcome the impact of other factors on lymph node detection. We looked into the long-term prognostic effectiveness of the three distinct lymph node stratification techniques for OS and CSS in patients. The most vital predicted efficacy criteria were used in developing the nomograms. A nomogram was used to determine each patient’s risk scores, and reliable risk score stratification of rectal cancer patients was used to perform prognostic analyses. Lastly, we discuss the advantages of radiation and chemotherapy for patients in various risk categories. This research aims to maximize the therapeutic benefit for patients by developing individualized treatment programs, accurately predicting long-term survival, and conducting a thorough investigation of various risk variables.

## Methods

### Inclusion of Patients

The data in this study were based on the SEER database, which included 8700 patients who were pathologically diagnosed with rectal cancer (209) or rectosigmoid junction cancer (199) according to the International Classification of Diseases of Oncology, Third Edition (ICD-O-3) from 2010 to 2015, and excluded patients who had incomplete basic clinical information due to the following reasons: TNM staging or AJCC staging was missing or unknown; the number of detected lymph nodes or positive lymph node counts were missing or unknown; survival information was missing; and tumor size was unknown. Based on the abovementioned criteria, 8700 patients were selected in this study. Subsequently, they were randomly divided into a training and validation cohorts with a ratio of 7:3. COX regression analysis was used to screen for significant risk factors so that line plots predicting 1-year, 3-year, and 5-year OS and CSS could be created.

### Variable Selection

Variables for the analysis conducted in this study included age, sex, histological grading, AJCC stage (7th), T stage, N stage, M stage, chemotherapy, radiotherapy, marital status, regional lymph nodes examined, regional lymph node positivity, survival status, months of survival, cause-specific death status, LNR, and LODDS. The formula for calculating LNR and LODDS based on the number of regional lymph nodes examined and the number of regional lymph node positives is as follows: LNR = regional lymph node positives / regional lymph nodes examined; LODDS = LOG (number of regional lymph node positives + 0.5 / number of regional lymph nodes examined − regional lymph node positives + 0.5). The optimum critical values of LNR and LODDS were calculated using X-tile software based on the principle of maximum rank square value and minimum *p* value. LNR was divided into three groups: LNR1 (0–0.048), LNR2 (0.049–0.278), and LNR3 (0.280–1). LODDS was divided into three groups: LODDS1 (− 2.25–1.301), LODDS2 (− 1.300–0.355), and LODDS3 (− 0.350–1.886).

### Statistical Analysis

The R software (version 4.22, https://www.r-project.org/) was used for all statistical analyses. All tests were two-sided, and *p* ≤ 0.05 was deemed statistically significant. Continuous data were expressed as mean ± standard deviation and median (range) and compared using the *t* tests or nonparametric tests. Categorical data were expressed as frequency (percentage) and compared with the chi-squared or Fisher’s exact tests. The N stage, LNR, and LODDS prediction performance for OS and CSS were compared using the *C* index, Akaike information criterion (AIC), and area under the receiver operating characteristic curve (AUC). The validity of N staging, LNR, and LODDS for prognostic classification of patients with rectal cancer was evaluated using Kaplan-Meier curves. Multivariate Cox analysis was used to identify independent risk factors for rectal cancer, and univariate Cox regression analysis was used to screen for prognosis-related risk factors. Hazard ratio (HR) with their 95% confidence intervals (CIs) were calculated. Recess cancer patients’ nomograms were created using the multifactorial analysis results to forecast OS and CSS at 1, 3, and 5 years. The *C* index, AUC, AIC, calibration curves, and DCA analysis were utilized to validate the nomograms’ predictive performance. The net benefit of the predictive models for OS, CSS, and other domains was compared with the AJCC staging system at various times. X-tile software performed risk stratification into three stages (NSL, NSM, and NSH). Kaplan-Meier analysis was then utilized to examine the variations in survival at each stage and further analyze the effects of the various risk stratifications on chemotherapy and radiation therapy.

## Results

### Patient Clinical Information

A total of 8700 rectal cancer patients were included in this study and were randomly assigned into training and validation cohorts with a ratio of 7:3. Specific clinical information and pathological characteristics are shown in Table 1.

**Table 1:** General clinical data for the training and validation cohorts

	**Training**	**Validation**	***p* value**
	**(*N* = 6090)**	**(*N* = 2610)**	
**Sex**			
Female	2524 (41.4%)	1061 (40.7%)	0.506
Male	3566 (58.6%)	1549 (59.3%)	
**Age**			
> 50 years	5049 (82.9%)	2163 (82.9%)	0.995
≤ 50 years	1041 (17.1%)	447 (17.1%)	
**Grade**			
I	368 (6.0%)	159 (6.1%)	0.71
II	4523 (74.3%)	1905 (73.0%)	
III	698 (11.5%)	318 (12.2%)	
IV	131 (2.2%)	55 (2.1%)	
Unknown	370 (6.1%)	173 (6.6%)	
**AJCC stage**			
I	1265 (20.8%)	558 (21.4%)	0.832
II	1591 (26.1%)	694 (26.6%)	
III	2586 (42.5%)	1087 (41.6%)	
IV	648 (10.6%)	271 (10.4%)	
**T stage**			
T1	673 (11.1%)	303 (11.6%)	0.732
T2	1054 (17.3%)	450 (17.2%)	
T3	3663 (60.1%)	1543 (59.1%)	
T4	700 (11.5%)	314 (12.0%)	
**N stage**			
N0	2985 (49.0%)	1306 (50.0%)	0.54
N1	2180 (35.8%)	929 (35.6%)	
N2	925 (15.2%)	375 (14.4%)	
**M stage**			
M0	5443 (89.4%)	2339 (89.6%)	0.766
M1	647 (10.6%)	271 (10.4%)	
**Radiation**			
None/unknown	3031 (49.8%)	1304 (50.0%)	0.888
Yes	3059 (50.2%)	1306 (50.0%)	
**Chemotherapy**			
No/unknown	2129 (35.0%)	947 (36.3%)	0.246
Yes	3961 (65.0%)	1663 (63.7%)	
**Tumor size**			
< 2 cm	733 (12.0%)	351 (13.4%)	0.075
> 5 cm	1844 (30.3%)	741 (28.4%)	
2–5 cm	3513 (57.7%)	1518 (58.2%)	
**LODDS**			
LODDS1	3200 (52.5%)	1392 (53.3%)	0.419
LODDS2	2272 (37.3%)	938 (35.9%)	
LODDS3	618 (10.1%)	280 (10.7%)	
**Marital status**			
Married	3497 (57.4%)	1531 (58.7%)	0.257
Other	1574 (25.8%)	679 (26.0%)	
Single	1019 (16.7%)	400 (15.3%)	

*LODDS* log ratio of positive lymph nodes

### Comparison of Predictive Efficacy of Different Lymph Node Analyses

**Fig. 1:**
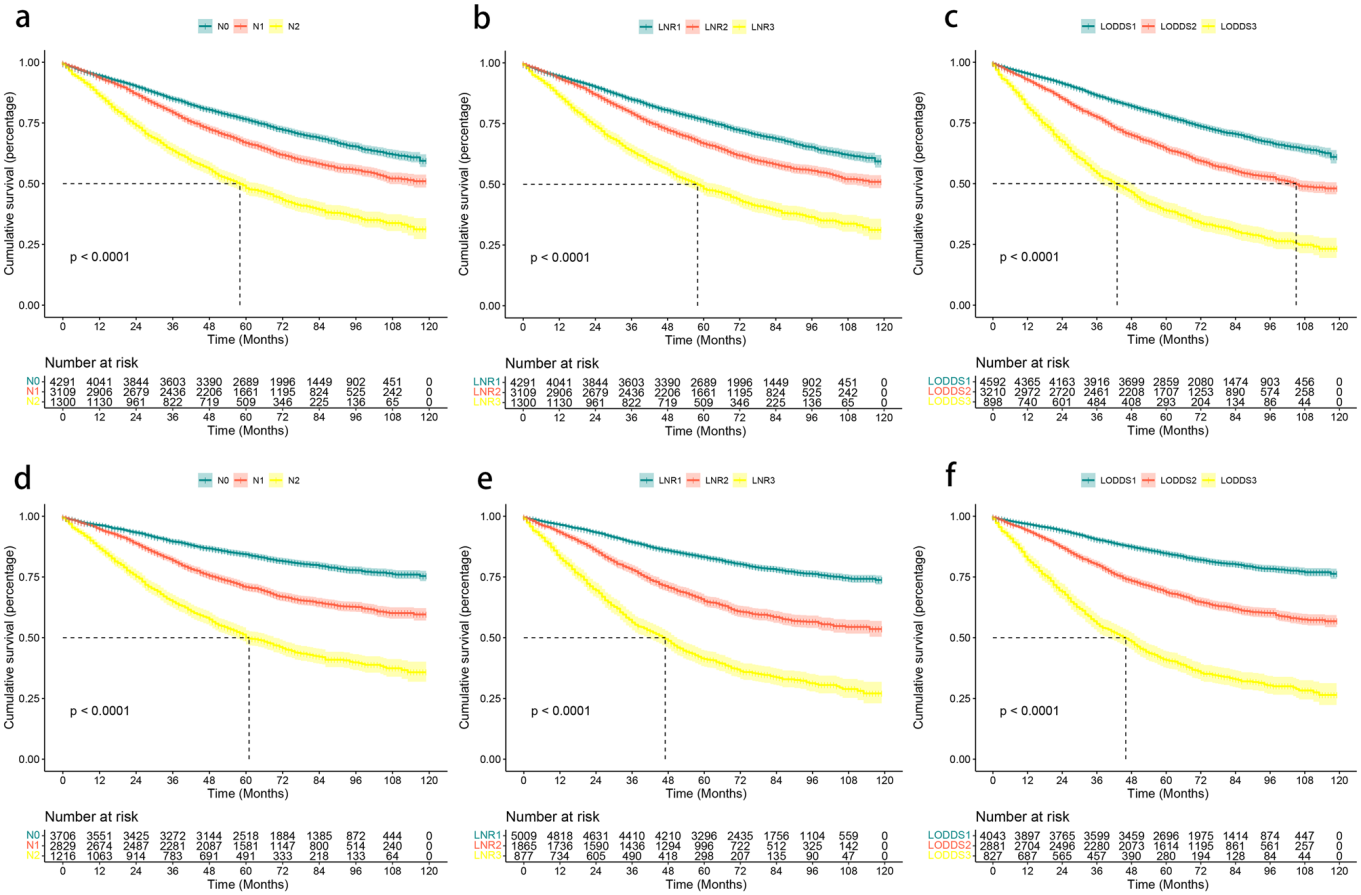
Kaplan-Meier analysis of overall survival (OS) and cancer-specific survival (CSS) of rectal cancer patients based on different lymph node staging modalities OS: **a** Kaplan-Meier curves based on N staging;** b** Kaplan-Meier curves based on LNR staging; and **c** Kaplan-Meier curves based on LODDS staging. CSS:** d** Kaplan-Meier curves based on N staging;** e** Kaplan-Meier curves based on LNR staging; and **f** Kaplan-Meier curves based on LODDS staging

Among the cohort of all patients, we firstly conducted a univariate COX regression analysis to evaluate the impact of various lymph node staging on overall prognosis. The findings indicated that N staging, LNR, and LODDS were significantly associated with (*p* < 0.001) patients with rectal cancer (Table 2). In addition, the three lymph node staging methods exhibited high predictive efficiency among different risk strata, as demonstrated by the Kaplan-Meier survival analysis (Fig. 1). The results show that LODDS staging has the highest AUC and *C* index values and the lowest AIC value (Table 3). This suggests that LODDS is more reliable than N staging and LNR in predicting the long-term survival outcome and has the best efficacy in predicting OS and CSS in patients with rectal cancer.

**Table 2:** Prognostic univariate COX regression analysis of three lymph node analyses

**Variable**		**OS**		**CSS**	
		**Hazard rate (95% CI)**		**Hazard rate (95% CI)**	***p* value**
N stage					
N0		Reference			
N1		1.413 (1.307–1.528)	< 0.001***	1.903 (1.727–2.097)	< 0.001***
N2		2.528 (2.311–2.766)	< 0.001***	3.82 (3.437–4.246)	< 0.001***
LNR					
LNR1		Reference			
LNR2		1.637 (1.511–1.774)	< 0.001***	2.171 (1.975–2.386)	< 0.001***
LNR3		3.235 (2.953–3.543)	< 0.001***	4.582 (4.133–5.079)	< 0.001***
LODDS					
LODDS1		Reference			
LODDS2		1.667 (1.546–1.799)	< 0.001***	2.169 (1.975–2.382)	< 0.001***
LODDS3		3.562 (3.234–3.923)	< 0.001***	5.23 (4.679–5.845)	< 0.001***

*p < 0.05; **p < 0.01; ***p < 0.001

**Table 3:** Comparison of prognostic efficacy of different lymph node staging criteria

**Variable**	**AIC**		***C* index**		**OS**			**CSS**		
	**OS**	**CSS**	**OS**	**CSS**	**1 year**	**3 years**	**5 years**	**1 year**	**3 years**	**5 years**
N stage	56,714	39,822	0.588	0.633	0.588	0.605	0.611	0.632	0.648	0.659
LNR stage	56,516	39,638	0.603	0.644	0.618	0.626	0.627	0.661	0.668	0.670
LODDS stage	56,490	39,626	0.610	0.649	0.628	0.634	0.633	0.665	0.672	0.673

*AIC* Akaike information criterion, *C index* concordance index

### Univariate and Multivariate COX Regression Analyses of Prognostic Factors

Univariate Cox regression analysis revealed that age, gender, histological stage, T stage, M stage, radiotherapy, chemotherapy, tumor size, marital status, and LODDS as risk factors for OS and CSS. Multifactorial analysis was performed, showing age, gender, histological staging, T staging, M staging, chemotherapy, tumor size, marital status, and LODDS as independent risk factors for OS (*p* < 0.05) (Table 4). There was a statistically significant difference (*p* < 0.05) in CSS concerning age, gender, histological staging, T staging, M staging, chemotherapy, marital status, and LODDS (Table 5).

**Table 4:** Univariate and multivariate COX regression analyses for OS

**Variable**	**OS**			
	**Univariate analysis**			**Multivariate analysis**
	**Hazard rate (95% CI)**	***p* value**	**Hazard rate (95% CI)**	***p* value**
Age				
≤ 50 years	Ref			
> 50 years	1.51866 (1.371–1.682)	< 0.001***	1.54207 (1.3893–1.712)	< 0.001***
Sex				
Female	Ref			
Male	1.146 (1.069–1.230)	< 0.001***	1.23011 (1.1446–1.322)	< 0.001***
Grade				
I	Ref			
II	1.04594 (0.8984–1.218)	0.563	0.95227 (0.8171–1.110)	0.531
III	1.72541 (1.4556–2.045)	< 0.001***	1.23388 (1.0383–1.466)	0.017*
IV	2.08907 (1.6405–2.660)	< 0.001***	1.45024 (1.1364–1.851)	0.003**
Unknown	0.86059 (0.6963–1.064)	0.165	0.88293 (0.7130–1.093)	0.254
T stage				
T1	Ref			
T2	1.5361 (1.293–1.824)	< 0.001***	1.60296 (1.3380–1.920)	< 0.001***
T3	2.38568 (2.054–2.771)	< 0.001***	2.56745 (2.1638–3.046)	< 0.001***
T4	4.84759 (4.115–5.710)	< 0.001***	4.05475 (3.3540–4.902)	< 0.001***
M stage				
M0	Ref			
M1	3.68658 (3.385–4.015)	< 0.001***	2.80166 (2.5480–3.081)	< 0.001***
Chemotherapy				
No/unknown	Ref			
Yes	0.8455 (0.7879–0.9074)	< 0.001***	0.46034 (0.4155–0.510)	< 0.001***
Radiation				
No/unknown	Ref			
Yes	0.74149 (0.6923–0.7942)	< 0.001***	1.01583 (0.9247–1.116)	0.743
Tumor size				
< 2 cm	Ref			
2–5 cm	1.4793 (1.305–1.676)	< 0.001***	1.01872 (0.8888–1.168)	0.79
> 5 cm	2.15466 (1.893–2.452)	< 0.001***	1.2149 (1.0502–1.405)	0.009**
Marital status				
Married	Ref			
Single	1.25604 (1.140–1.384)	< 0.001***	1.21621 (1.1033–1.341)	< 0.001***
Other	1.66198 (1.539–1.794)	< 0.001***	1.57651 (1.4565–1.706)	< 0.001***
LODDS				
LODDS1	Ref			
LODDS2	1.66725 (1.546–1.799)	< 0.001***	1.64321 (1.5173–1.780)	< 0.001***
LODDS3	3.56171 (3.234–3.923)	< 0.001***	2.89328 (2.6024–3.217)	< 0.001***

*p < 0.05; **p < 0.01; ***p < 0.001

**Table 5:** Univariate and multifactor COX regression analyses for CSS

**Variable**	**CSS**			
	**Univariate analysis**			**Multivariate analysis**
	**Hazard rate (95% CI)**	***p* value**	**Hazard rate (95% CI)**	***p* value**
Age				
≤ 50 years	Ref			
> 50 years	1.234 (1.106–1.377)	< 0.001***	1.3531 (1.2095–1.5138)	< 0.001***
Sex				
Female	Ref			
Male	1.109 (1.021–1.205)	0.014*	1.166 (1.0713–1.2691)	< 0.001***
Grade				
I	Ref			
II	1.1927 (0.9815–1.449)	0.076	1.0105 (0.8307–1.2291)	0.917
III	2.2689 (1.8360–2.804)	< 0.001***	1.3758 (1.1104–1.7046)	0.004*
IV	2.7141 (2.0426–3.606)	< 0.001***	1.6378 (1.2292–2.1822)	< 0.001***
Unknown	0.9562 (0.7350–1.244)	0.739	0.8565 (0.6570–1.1165)	0.252
T stage				
T1	Ref			
T2	1.854 (1.440–2.387)	< 0.001***	2.0029 (1.5416–2.6021)	< 0.001***
T3	3.928 (3.149–4.900)	< 0.001***	3.8257 (2.9940–4.8883)	< 0.001***
T4	9.186 (7.288–11.578)	< 0.001***	6.4824 (4.9930–8.4160)	< 0.001***
M stage				
M0	Ref			
M1	4.874 (4.874–5.348)	< 0.001***	3.1792 (2.8664–3.5260)	< 0.001***
Chemotherapy				
No/unknown	Ref			
Yes	1.108 (1.014–1.211)	0.023*	0.4958 (0.4399–0.5589)	< 0.001***
Radiation				
No/unknown	Ref			
Yes	0.826 (0.7616–0.8958)	< 0.001***	1.0437 (0.9395–1.1595)	0.425
Tumor size				
< 2 cm	Ref			
2–5 cm	1.632 (1.395–1.909)	< 0.001***	0.9169 (0.7748–1.0850)	0.312
> 5 cm	2.619 (2.232–3.075)	< 0.001***	1.1355 (0.9513–1.3554)	0.159
Marital status				
Married	Ref			
Single	1.2849 (1.206–1.506)	< 0.001***	1.2692 (1.1488–1.4370)	< 0.001***
Other	1.5582 (1.495–1.798)	< 0.001***	1.5614 (1.4168–1.7136)	< 0.001***
LODDS				
LODDS1	Ref			
LODDS2	2.169 (1.975–2.382)	< 0.001***	1.9461 (1.7638–2.1472)	< 0.001***
LODDS3	5.23 (4.679–5.845)	< 0.001***	3.6024 (3.1893–4.0691)	< 0.001***

*p < 0.05; **p < 0.01; ***p < 0.001

### Construction and Validation of the OS and CSS Nomograms

**Fig. 2:**
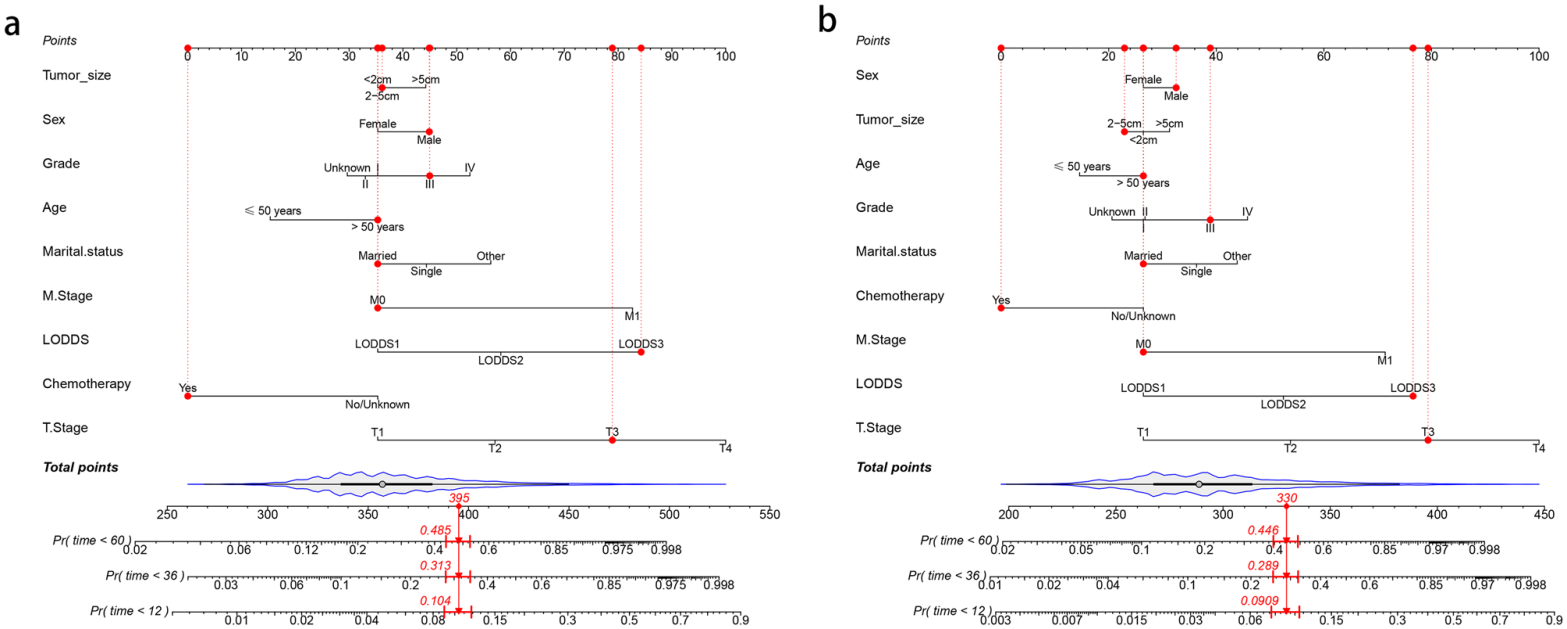
Training cohort-based construction of prognostic nomograms predicting 1-, 3-, and 5-year prognosis in patients with rectal cancer: **a** nomograms predicting overall survival (OS) and **b** nomograms predicting cancer-specific survival (CSS)

**Fig. 3:**
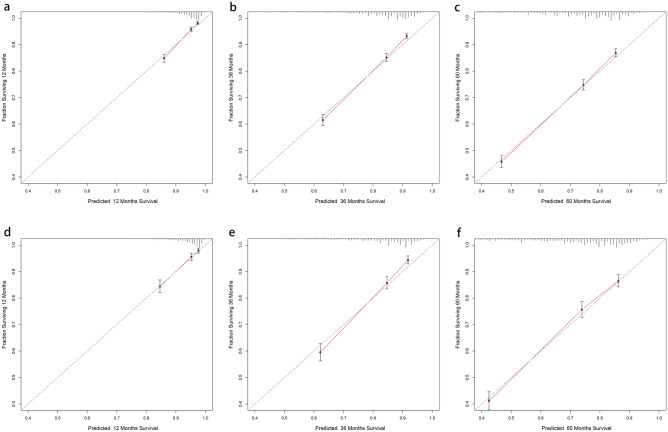
Correction curves for nomograms constructed on the basis of overall survival (OS). **a**–**c** Correction curves for the training cohort at 1, 3, and 5 years, in that order. **d**–**f** Correction curves for the validation cohort at 1, 3, and 5 years, in that order

**Fig. 4:**
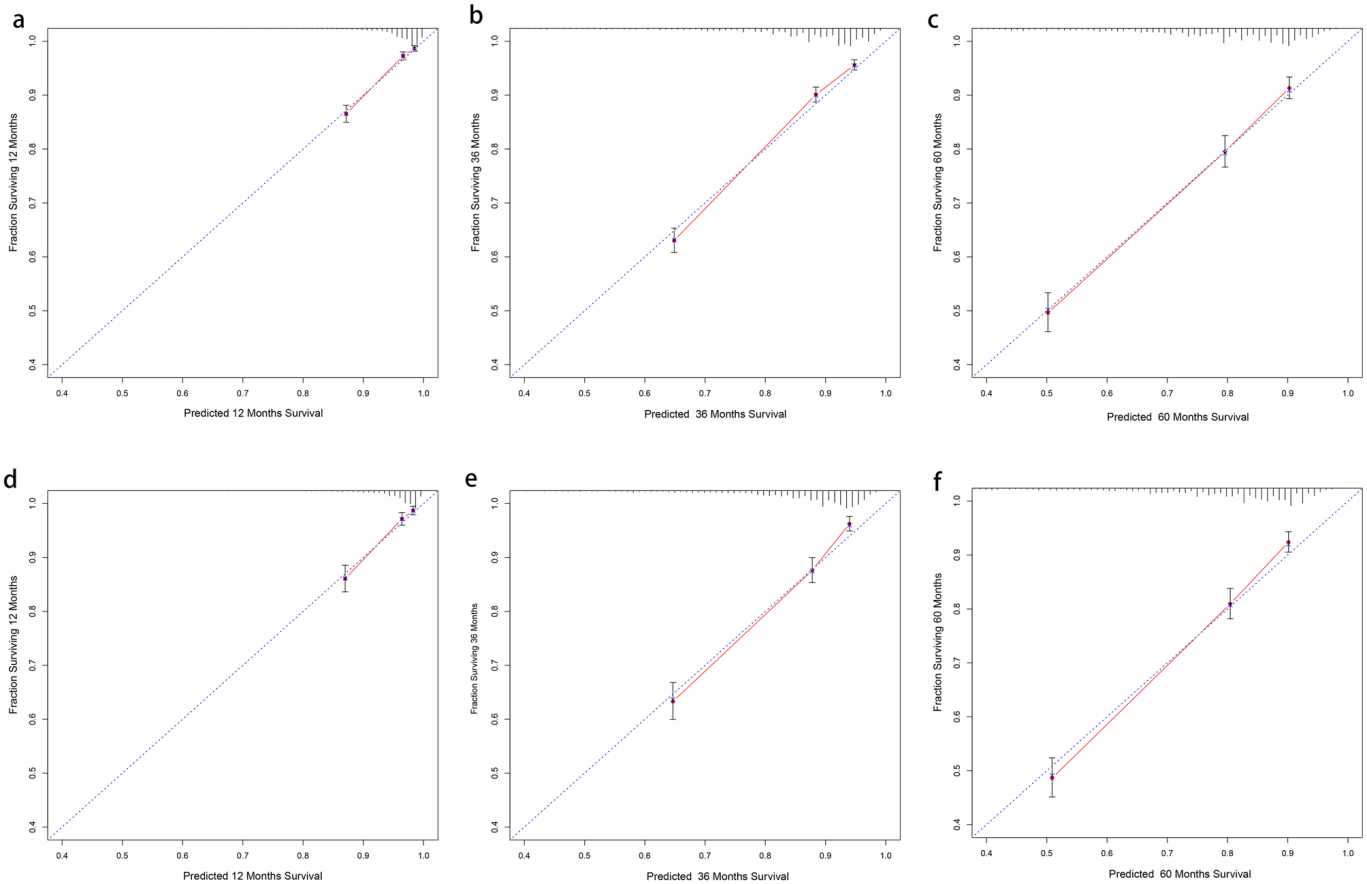
Correction curves for nomograms constructed based on cancer-specific survival (CSS). **a**–**c** Correction curves for the training cohort at 1, 3, and 5 years, in that order. **d**–**f** Correction curves for the validation cohort at 1, 3, and 5 years, in that order

Based on the results of the above analysis, we selected 9 variables, including LODDS (age, gender, histological grading, T staging, M staging, tumor size, chemotherapy, LODDS, marital status) to construct OS and CSS prognostic nomograms for rectal cancer patients (Fig. 2). The patient’s OS and CSS were predicted by aggregating the relative risk scores of the patient’s risk variables to get a final total risk score. The calibration curves were close to the standard curves in both the training and validation cohorts, indicating that the column plot had exceptional predictive accuracy in predicting 1-, 3-, and 5-year OS and CSS (Figs. 3 and 4).

### Prognosis of Patients with Rectal Cancer by the Nomograms Versus Conventional AJCC Staging

**Fig. 5:**
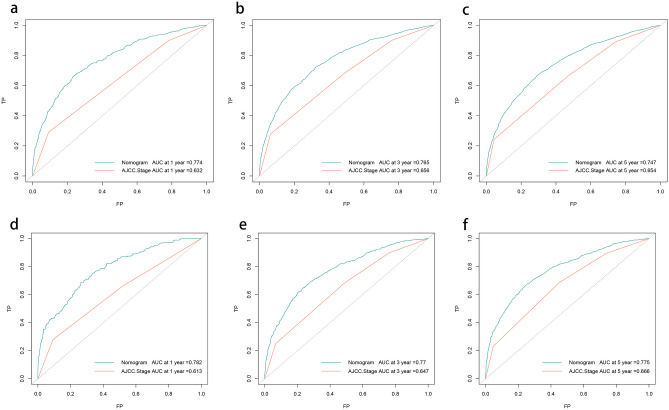
Receiver operating characteristic curve (ROC) based on nomograms constructed for overall survival (OS). **a**–**c** ROC curves for the training cohort at 1, 3, and 5 years, in that order. **d**–**f** ROC curves for the validation cohort at 1, 3, and 5 years, in that order

**Fig. 6:**
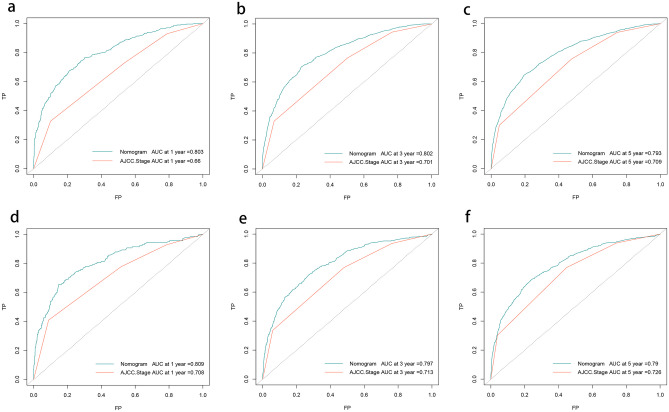
Receiver operating characteristic curve (ROC) based on nomograms constructed for cancer-specific survival (CSS).** a–c** ROC curves for the training cohort at 1, 3, and 5 years, in that order; **d–f** ROC curves for the validation cohort at 1, 3, and 5 years, in that order

**Fig. 7:**
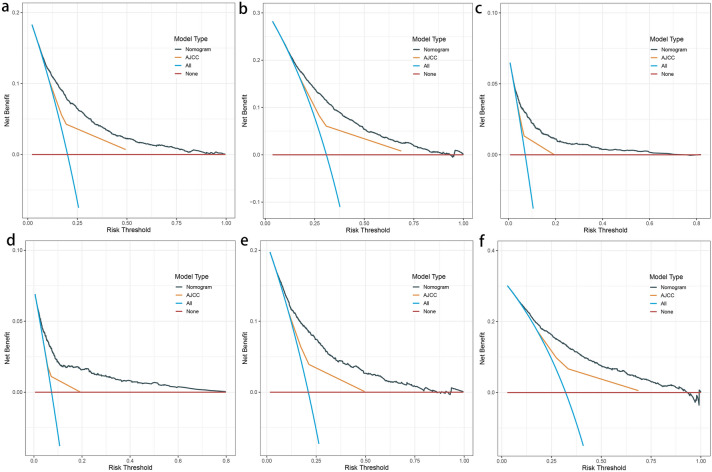
Decision curve analysis (DCA) based on the nomograms constructed for overall survival (OS). **a**–**c** DCA at 1, 3, and 5 years for the training cohort in order. **d**–**f** DCA at 1, 3, and 5 years for the validation cohort in order

**Fig. 8:**
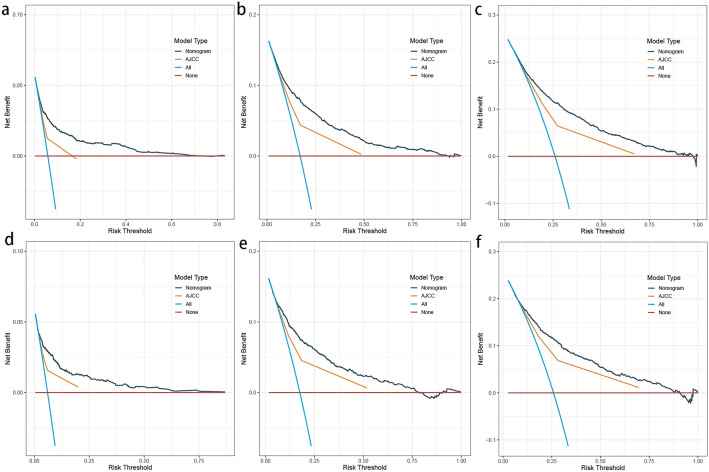
Decision curve analysis (DCA) constructed based on cancer-specific survival (CSS). **a**–**c** DCA at 1, 3, and 5 years for the training cohort in order. **d**–**f** DCA at 1, 3, and 5 years for the validation cohort in order

The *C* indices of the LODDS-based nomograms for OS and CSS for the training and validation cohorts, respectively (OS training cohort 0.716, validation cohort 0.726; CSS training cohort 0.755, validation cohort 0.757) were significantly higher than those for the traditional AJCC staging (OS training cohort 0.624, validation cohort 0.629; CSS training cohort 0.679, validation cohort 0.678). Meanwhile, the nomograms of AIC (OS training cohort 36,625, validation cohort 14,441; CSS training cohort 25,666, validation cohort 9877) were lower than the AJCC staging (OS training cohort 37,390, validation cohort 14,797 CSS; training cohort 26,278, validation cohort 10,121) (Table 6). The ROC curves also showed good discriminatory ability and predictive accuracy (Figs. 5 and 6). The DCA curves showed that the nomograms constructed based on the LODDS would achieve a more significant net gain than traditional AJCC staging, which is more favorable to the clinical benefit of rectal cancer patients (Figs. 7 and 8).

**Table 6:** Comparison of prognostic efficacy of the nomograms and AJCC staging for long-term prognosis

	**AIC**				***C* index**			
	**TRAIN**		**Validation**		**TRAIN**		**Validation**	
	**Nomogram**	**AJCC**	**Nomogram**	**AJCC**	**Nomogram**	**AJCC**	**Nomogram**	**AJCC**
OS	36,625	37,390	14,441	14,797	0.716	0.624	0.726	0.629
CSS	25,666	26,278	9877	10,121	0.755	0.679	0.757	0.678

*AIC* Akaike information criterion, *C index* concordance index

### Survival Curves and Adjuvant Treatment Effects Based on the Nomograms Risk Scores

**Fig. 9:**
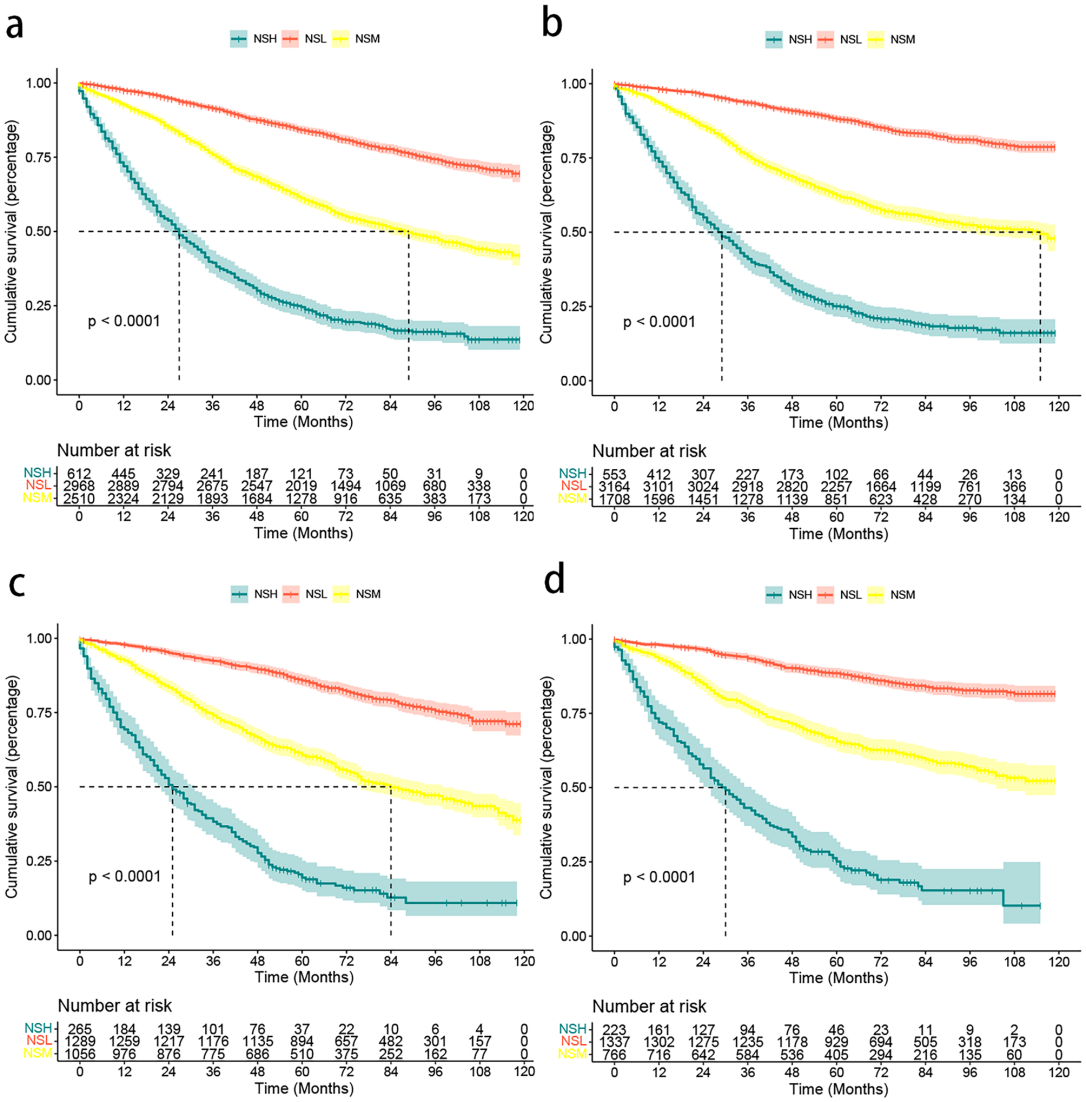
Kaplan-Meier survival analyses based on the nomograms’ hazard scores for nomogram high-scoring group (NSH), nomogram medium-scoring group (NSM), and nomogram low-scoring group (NSL). **a** Kaplan-Meier curves in the OS training set. **b** Kaplan-Meier curves in the CSS training set. **c** Kaplan-Meier curves in the OS validation set. **d** Kaplan-Meier curves in the CSS validation set

We calculated the total risk scores for OS and CSS for each patient based on the nomograms using R language calculations, respectively, and risk-stratified them using the X-tile software into a high-risk group (NSH, OS 242.01 < NSH < 411.19; CSS 208.43 < NSH < 348.49), an intermediate risk group (NSM, OS 160.25 < NSM < 241.87, CSS 141.05 < NSM < 208.38), and low-risk group (NSL, OS NSL < 160.19, CSS NSL < 141.04), and then, Kaplan-Meier analyses were carried out on them. Based on our risk score stratification, the results showed that OS or CSS had good differentiation between high-risk, moderate-risk, and low-risk insurance groups (Fig. 9). We then further explored the impact of chemotherapy on patient survival according to different risk stratifications. In the high-risk group, chemotherapy showed a significant advantage in improving patients’ CSS and OS survival, and the intermediate-risk group showed a similar trend, but the results were not statistically significant. In the low-risk group, however, chemotherapy demonstrated deleterious effects on OS and CSS (Supplementary Fig. 1). Similar results to chemotherapy were demonstrated at the radiotherapy level, with a significant benefit at the radiotherapy level in the high-risk group. However, a similar trend towards benefit was demonstrated in the intermediate-risk group and a trend towards detriment to radiotherapy in the low-risk group. However, no statistically reliable results were obtained in either group (Supplementary Fig. 2).

## Discussion

Lymph node status is an essential factor affecting local recurrence and overall survival in patients with rectal cancer [15]. Treatment options regarding rectal cancer are increasingly converging towards surgery combined with radiotherapy and chemotherapy [16, 17], and the pathological staging of lymph nodes is closely related to adjuvant treatment options. Currently, AJCC staging is widely used to evaluate the pathological staging of lymph nodes. However, this criterion is affected by various factors such as surgical approach, pathological assessment, adjuvant therapy, and BMI of the patient, compromising the precision of staging the pathology [6, 18]. In recent years, the emergence of LNR and LODDS has been recognized as a better alternative to traditional N staging. It is not limited by the number of lymph nodes detected, reducing staging migration [19, 20]. Several studies have confirmed that LODDS is an independent prognostic factor for different types of cancers and has a more accurate predictive efficacy than traditional AJCC staging and LNR [21–23].

Through univariate COX regression analysis, we can see that the N, LNR, and LODDS stages are all potential prognostic factors for patients with rectal cancer. The Kaplan-Meier survival curves also revealed that the three staging schemes were able to stratify the patients’ prognostic status and that the LODDS staging had the highest *C* index, AUC value, and lowest AIC value when compared to the N staging and LNR staging, indicating that it was the most effective at predicting the prognosis of patients with rectal cancer. In univariate and multivariate COX regression analysis of prognostic factors in the patient population, the other risk factors affecting the prognosis of patients with rectal cancer include age, gender, histological grading, T staging, M staging, chemotherapy, tumor size, LODDS, and marital status. Based on our constructed nomograms, the predictive efficacy of 1-, 3-, and 5-year OS and CSS for rectal cancer patients was significantly higher than that of AJCC staging. It was more pronounced in the training cohorts and validation cohorts of CSS. The correction curves likewise showed good agreement with the actual observations. Guo et al. explored that LODDS had predicted CSS in T1 stage rectal cancer [24], showing satisfactory results, and within T1 stage, LODDS can be further stratified for more accurate survival prediction and identification of high-risk patients. However, its applicability in other stages has to be confirmed. Christina et al. predicted survival in patients with stage III rectal cancer [5]. However, the sample size of this study was too small, except for staging limitations, and there was a higher likelihood of class II error. The present study has no strict limitations on pathological staging, has good applicability, and included 8700 patients with rectal cancer, making it a reliable study. It is worth mentioning that tumor size did not show statistically significant in the CSS cohort. The reason may be attributed to unclear patient information in some of the SEER databases and other reasons for the impact of patient death on survival status. However, studies have shown that tumor size is closely associated with long-term survival in colorectal cancer, with a prognostic value of clinical importance no less than that of the T staging [25–27]. Therefore, we have included it in the CSS the nomograms.

Currently, adjuvant treatment options for rectal cancer remain controversial [28], with some articles reporting no significant improvement in survival benefits with adjuvant chemotherapy in older patients with stage II and III rectal cancer [29–31], and an increase in chemotherapeutic complications as well as toxic effects [32, 33]. Therefore, identifying the population that will benefit from adjuvant therapy is crucial. Until now, no studies have been found to explore the correlation between LODDS and adjuvant therapy for rectal cancer. We scored the included patients’ basic clinical information and pathological characteristics using a nomogram. We used X-tile software to categorize the patients in the training cohort into three groups based on their nomogram scores: a nomogram high-scoring group, a nomogram medium-scoring group, and a nomogram low-scoring group. Regarding chemotherapy, the high-scoring group showed a significant survival benefit, with a statistically significant difference (*p* < 0.05).

On the contrary, the low-scoring group demonstrated a detrimental trend. A beneficial trend was also demonstrated in the nomogram medium-scoring group. However, statistical significance has yet to be reached in the validation group, and it is reasonable to infer that it is related to insufficient sample size in the training cohort. In radiotherapy, similar to chemotherapy, only the nomogram high-scoring group showed improved survival outcomes, with the nomogram medium-scoring group and the nomogram low-scoring group showing detrimental trends. These results suggest our nomograms, which are essential for the treatment of rectal cancer patients. There are some limitations of this study; firstly, the cohort lacked an external validation cohort to confirm further the model’s predictive ability for OS and CSS. Secondly, the clinical data included in this study inevitably suffered from retrospective bias, with some patients needing more clinical information, resulting in their non-inclusion in the study cohort. Future large-scale prospective clinical data are needed to confirm the reliability of the findings of this study.

## Conclusion

This study is based on the population to develop the LODDS-based nomograms, which were confirmed to have superior predictive ability by analysis. And LODDS is a more reliable prognostic predictor than conventional TNM staging and LNR. The nomogram-based prognostic classification is valuable for identifying high-risk populations that may benefit from adjuvant treatment. In summary, these nomograms can be a reliable prognostic predictor for rectal cancer patients and can help guide patient treatment.

### Supplementary Information

Below is the link to the electronic supplementary material.

Supplementary file1 (DOCX 1074 KB)

## Data Availability

No datasets were generated or analysed during the current study.
