# Supplementary material for: A Prognostic Model Based on the Log Odds Ratio of Positive Lymph Nodes Predicts Prognosis of Patients with Rectal Cancer
Source: J Gastrointest Cancer. 2024 May 3;55(3):1111–24. doi: 10.1007/s12029-024-01046-2 (PMC11347484; doi:10.1007/s12029-024-01046-2)

Supplementary figure 1. Survival impact of chemotherapy within each risk stratum: **(a-c)** survival curves for nomogram high scoring group (NSH), nomogram medium scoring group (NSM), and nomogram low scoring group (NSL) based on OS risk scores in the training cohort, in order; **(d-f)** survival curves for NSH, NSM, and NSL populations based on OS risk scores in the validation cohort, in order. **(g-i)** Survival curves for NSH, NSM, and NSL populations in the training cohort based on CSS risk scores, in that order; **(j-l)** Survival curves for NSH, NSM, and NSL populations in the validation cohort based on CSS risk scores, in that order.


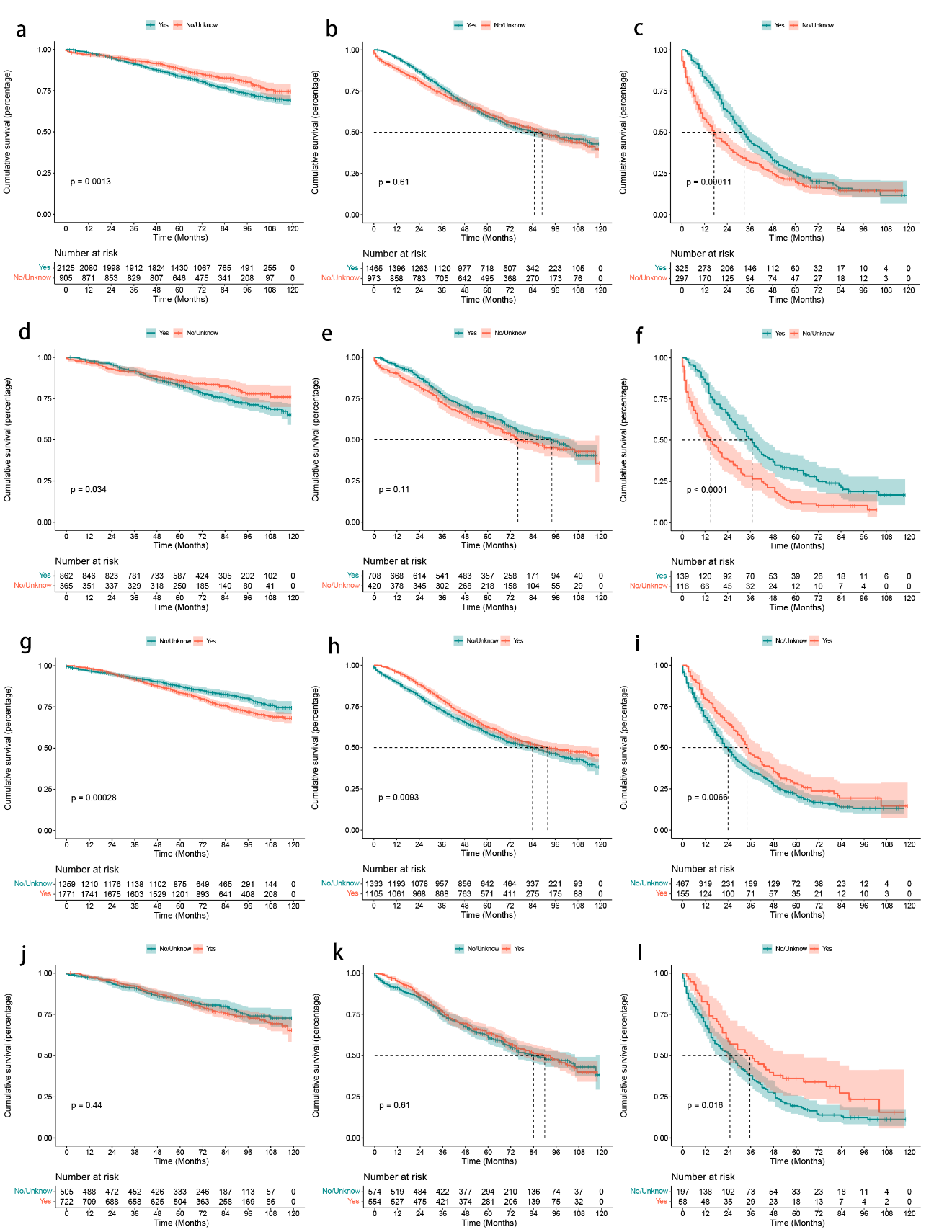


Supplementary figure 2. Survival analysis of radiotherapy within different risk strata: **(a-c)** survival curves for nomogram high scoring group (NSH), nomogram medium scoring group (NSM), and nomogram low scoring group (NSL) based on OS risk scores in the training cohort, in order; **(d-f)** survival curves for NSH, NSM, and NSL populations based on OS risk scores in the validation cohort, in order. **(g-i)** Survival curves for NSH, NSM, and NSL populations in the training cohort based on CSS risk scores, in that order; **(j-l)** Survival curves for NSH, NSM, and NSL populations in the validation cohort based on CSS risk scores, in that order.


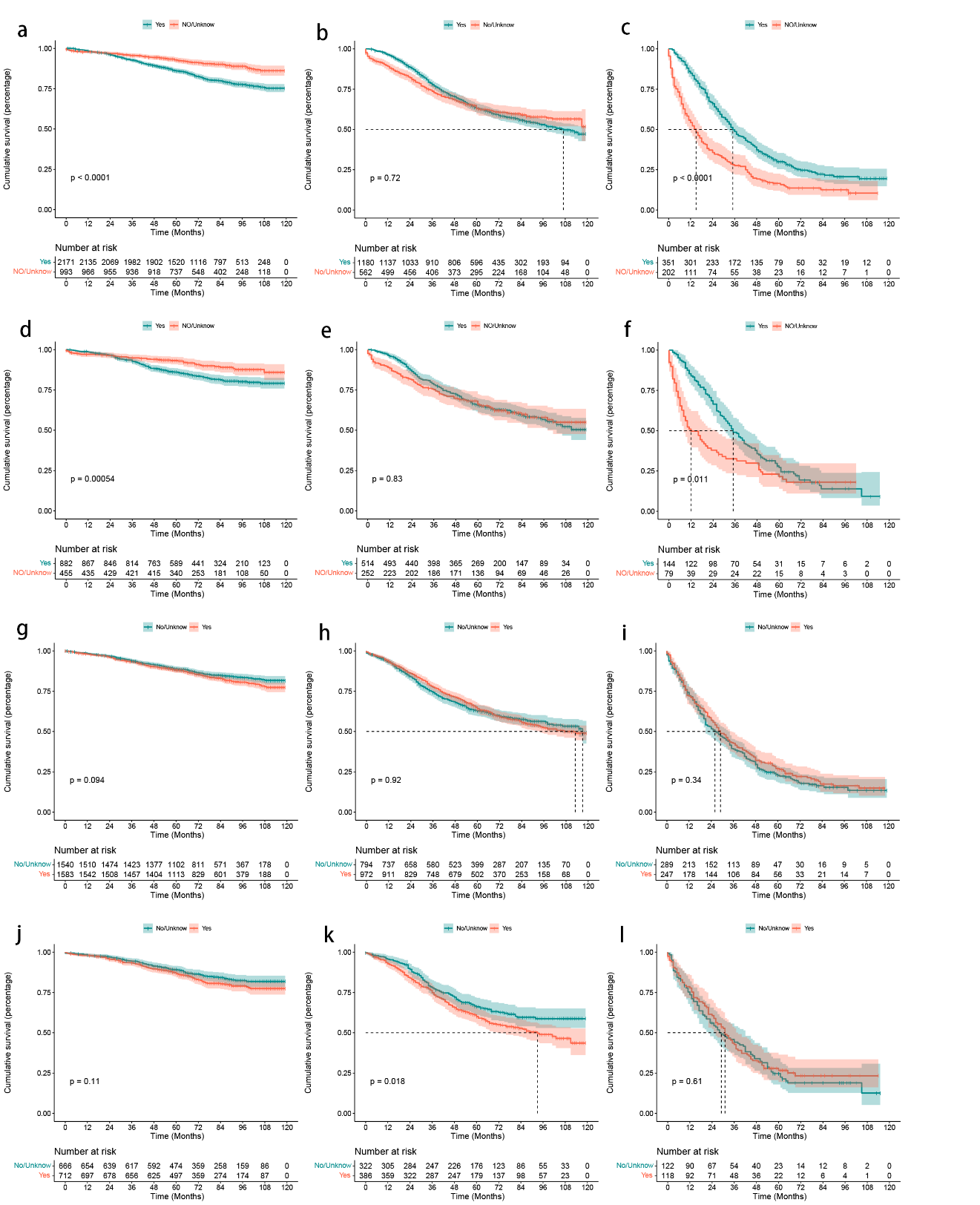

Supplement: Supplementary file 1 — Supplementary file1 (DOCX 1074 KB) [file 12029_2024_1046_MOESM1_ESM.docx]
